# Revisiting Mednick’s Model on Creativity-Related Differences in Associative Hierarchies. Evidence for a Common Path to Uncommon Thought

**DOI:** 10.1002/jocb.35

**Published:** 2013-11-15

**Authors:** Mathias Benedek, Aljoscha C Neubauer

**Affiliations:** 1 University of GrazAustria

**Keywords:** creativity, cognition, memory, attention, executive functions

## Abstract

Fifty years ago, Mednick [*Psychological Review*, 69 (1962) 220] proposed an elaborate model that aimed to explain how creative ideas are generated and why creative people are more likely to have creative ideas. The model assumes that creative people have flatter associative hierarchies and as a consequence can more fluently retrieve remote associative elements, which can be combined to form creative ideas. This study aimed at revisiting Mednick’s model and providing an extensive test of its hypotheses. A continuous free association task was employed and association performance was compared between groups high and low in creativity, as defined by divergent thinking ability and self-report measures. We found that associative hierarchies do not differ between low and high creative people, but creative people showed higher associative fluency and more uncommon responses. This suggests that creativity may not be related to a special organization of associative memory, but rather to a more effective way of accessing its contents. The findings add to the evidence associating creativity with highly adaptive executive functioning.

Observation and quantification of word associative behavior was among the first methods for the study of human behavior (e.g., Jung, 1910). Empirical research in this context commonly employs either single or continuous word association tests. Single word association tests (e.g., Kent & Rosanoff, 1910) present a list of words and ask for the primary association that comes to mind to each word in the list. Responses in this test can be analyzed for uncommonness by means of word association norms specifying their relative frequency of occurrence in large samples (Postman & Keppel, 1970). Moreover, the relative response frequency of association responses is also considered as an indicator of associative strength (Wettler, Rapp, & Sedlmeier, 2005). Such association measures have been applied in the study of semantic representations in memory (e.g., Nelson, McEvoy, & Dennis, 2000). Continuous word association tests ask for a continuous sequence of association responses which additionally allows studying temporal characteristics of associative behavior. This forms the empirical basis of various models of semantic processing including, for example, the dynamics of verbal clustering or free recall (e.g., Bousfield & Sedgewick, 1944; Raaijmakers & Shiffrin, 1981). From an individual differences perspective, association behavior was primarily studied in the context of mental illness and psychosis-prone personality. Deviating associative behavior was found to be related to factors such as mania, schizophrenia, schizotypy, and psychoticism (Giehm, 1933; Merten, 1993; Merten & Fischer, 1999; Rominger, Weiss, Fink, Schulter, & Papousek, 2011).

Fifty years ago, Mednick (1962) put forward an elaborate theoretical model in which he outlined the presumed relationship of associative behavior and creativity. Mednick defined the process of creative thinking as “the forming of associative elements into new combinations which either meet specific requirements or are in some way useful. The more mutually remote the elements of the new combination, the more creative the process or solution” (1962, p. 221). On the basis of this definition, he assumed that creative individuals show higher ability to access mutually remote associative elements, which then can be combined to form creative solutions. More specifically, Mednick proposed that creative people are characterized by flatter associative hierarchies as compared to less creative people who are characterized by steeper associative hierarchies (see Figure 1). Associative hierarchies refer to the idea that for any given concept there is a set of associations which can be arranged in the order of their associative strength. A person with steep associative hierarchies hence would have a couple of stereotypical associations with very high association strength, while for further associations the association strength and hence the probability of retrieval would be much lower (e.g., a person with steep associative hierarchies being presented the concept “table” might be restricted to overly dominant responses such as “chair”; see Figure 1). In contrast, a person with a flat associative hierarchy would show a less skewed distribution of association strengths with more subtle differences between different associations (e.g., a person with flat associative hierarchies being presented the concept “table” is more likely to retrieve more remote association responses such as “leg” or “food”; see Figure 1). This conceptual framework led him to the development of the Remote Associates Test (RAT; Mednick, 1968). In this test, participants are presented three unrelated words (e.g., “rat—blue—cottage”) and they have to find a forth word (e.g., “cheese”) which serves as an associative link for the stimulus words.

**Figure 1:**
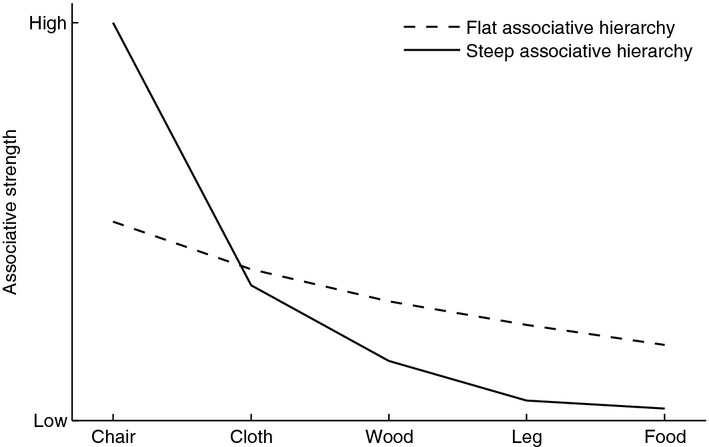
Association hierarchies for the concept “table” (adapted from Mednick, 1962, p. 223). According to Mednick, creative people show flatter associative hierarchies.

Mednick derived specific testable hypotheses from his model of associative hierarchies. First, he assumed that creative people (flat associative hierarchies) should generate associations initially at a lower rate due to the absence of highly dominant responses; but they should also respond more steadily and thus in the long run emit an overall higher number of responses. In other words, creative people should show higher associative fluency (i.e., a higher number of association responses per time), but this effect may interact with time. Second, the associations of creative people should be characterized by higher uncommonness (i.e., lower associative strength).

## Empirical Tests of Mednick’s Model

So far, there is no direct empirical test of Mednick’s (1962) main hypothesis comparing the associative hierarchies of high and low creative people. A number of studies, however, aimed at testing the hypotheses derived from Mednick’s model concerning fluency and uncommonness of associative behavior. We will first look at studies which focused on the presumed relation of creativity and associative fluency. Mednick, Mednick, and Jung (1964) employed a continuous word association task and studied the response rate over time comparing male participants scoring low, average, or high on the RAT. In line with the predictions, they found that high RAT scorers produced a higher number of associations per time; however, high RAT scorers responded more fluently at any time of the task, which stands in contrast with the original prediction of an initially slower response rate. Similarly, in a study by Piers and Kirchner (1971) especially female participants (but also male participants for certain types of stimuli) with high RAT scores showed increased associative fluency in a 2-min continuous word association task. Levin (1978) analyzed the clustering of responses during continuous association and found that high RAT scorers actually do not differ in the number of responses directly focusing on the stimulus word, but show higher fluency of chained associations (i.e., associations focusing on the preceding response rather than the stimulus word).

Other studies addressed the question whether creativity is related to more uncommon association behavior. Olczak and Kaplan (1969) compared the uncommonness of the first six association responses of RAT low and high scorers and found that the uncommonness of later responses was higher but did not differ between groups. Piers and Kirchner (1971) obtained the same finding using a continuous association task. Riegel, Riegel, and Levine (1966) devised a set of 13 constrained primary association tasks (i.e., finding synonyms, contrasts, subordinates,…) and compared responses of groups high or low in creative personality (Creative Personality Scale; Fricke, 1965). Their main finding was that creative personality was associated with a higher variability in responses (i.e., a higher number of nonredundant responses) in many of their tasks, whereas low creative personality was related to more stereotyped responses. Rothenberg (1973) studied the tendency of opposite responding during word association in groups of high and low self-reported creativity (assessed by a self-devised questionnaire on performance and motivation in scientific and artistic creativity). He found that high self-reported creativity was related to a faster and more reliable responding of opposites, especially for words that typically evoke opposites (e.g., “black” evokes the primary association “white” in 75%; of participants; Jenkins, 1970). Rothenberg attributed this finding to the creativity-relevant capacity of simultaneously conceiving opposite concepts (i.e., Janusian thinking). However, this particular finding of a faster responding of opposites (which usually have very high response strengths) would rather correspond to the definition of a steep associative hierarchy and thus be inconsistent with Mednick’s definition of creativity. Gough (1976) analyzed the primary associations of scientists and university students by classifying all responses into five categories representing decreasing frequency of occurrence. He found that individual creativity (assessed by peer- and supervisor ratings) was positively correlated with moderately infrequent responses (1–10%;), but neither with common (>10%;) nor extremely uncommon/unique (<1%;) responses. This finding hence points at a nonlinear relation of creativity and uncommonness of associations. In a more recent study, Merten and Fischer (1999) compared primary word associations of creative people (writers and actors) to a clinical group of schizophrenics and a control group. They found that creative people showed even more uncommon responses than schizophrenics when the task asked for free or uncommon responses, but when the task asked for common responses the creative group, in contrast to schizophrenics, did no longer differ from the control group. They concluded that creativity cannot be generally related to deviating associative behavior.

Finally, there are also studies which tried to test other predictions derived from Mednick’s (1962) model. Brown (1973) hypothesized that flatter associative hierarchies should facilitate the learning of weak associates. In line with Mednick’s model, he found that RAT high scorers showed a lower performance difference in learning lists of strongly versus weakly related words than RAT low scorers. Coney and Serna (1995) hypothesized that individual differences in the slope of associative hierarchies should result in a differential facilitation effect by primes of low, medium or high associative strength. This prediction, however, could not be supported. High creative people (Torrance Test of Creative Thinking; TTCT; Torrance, 1974) were less facilitated by primes at all levels of strength.

To summarize, the literature on creativity and word association behavior so far offers only partial support for Mednick’s (1962) model. Mednick assumed that creative people have flatter associative hierarchies and therefore should show higher associative fluency (at least in the long run), and higher uncommonness. The notion of higher associative fluency was supported by a number of studies (Levin, 1978; Mednick et al., 1964; Piers & Kirchner, 1971), but this effect may not interact with time (Mednick et al., 1964). Concerning the uncommonness of associations, the evidence is inconsistent, either reporting no relationship (Olczak & Kaplan, 1969; Piers & Kirchner, 1971), a positive relationship (Merten & Fischer, 1999; Riegel et al., 1966), a negative relationship (Rothenberg, 1973), or a nonlinear relationship (Gough, 1976) between associative uncommonness and individual creativity. Further tests of Mednick’s model also could not provide conclusive results (Brown, 1973; Coney & Serna, 1995).

## Aims of this Study

The inconsistent findings related to Mednick’s (1962) model may have different reasons. In part, the validity of results may be constrained by the predominant use of the RAT as a single and not indisputable criterion of individual creativity. It should be noted that the validity of the RAT as a measure of creativity has been repeatedly put into question (e.g., Mendelsohn, 1976; Worthen & Clark, 1971). And more importantly, testing the relationship between association behavior and creativity by means of the RAT, which in fact also attempts to assess specific associative abilities, reflects a serious conceptual confound. Moreover, studies differ in focusing either on a constant number of primary associations or on unrestricted continuous association behavior. Finally, so far no study made the attempt to explicitly test Mednick’s model directly by comparing the associative gradients related to high and low creativity.

This study, therefore, aims to revisit Mednick’s intuitively appealing ideas and provide an initial, direct test of his model and all related hypotheses. Groups of high and low creativity are defined by means of a representative set of commonly used indicators of creativity including self-report measures as well as tests of divergent thinking ability (cf., Kaufman, Plucker, & Baer, 2008). To explicitly test the assumption of differential associative hierarchies, the performance in a continuous free association test is used to map associative hierarchies separately for groups of high and low creativity. The gradients of associative hierarchies then are compared between groups. Mednick’s model would be supported if high creative people showed flatter associative hierarchies than low creative people (i.e., lower association strengths for dominant responses and higher association strengths for remote responses). Moreover, associative fluency and uncommonness are compared between creativity groups and analyzed over time with respect to constant periods of time, as well as with respect to constant number of responses (uncommonness only). Mednick’s model would predict that high creative people show higher associative fluency especially in the long run (interaction of associative fluency with time), and also higher uncommonness of association responses than low creative people.

## Method

### Participants

A total of 150 undergraduate students (42%; female participants) with an average age of 22 years (*SD*  =; 2.7) participated in this study. The sample comprised a wide range of majors which are very heterogeneous with respect to creativity-related demands such as industrial design, information design, industrial engineering or psychology. As compensation for participation, the participants were offered a brief report on their personality and creativity scores. All participants gave written informed consent.

### Materials and Procedure

The data were collected as a part of a larger study on individual differences in cognitive abilities. Participants completed a number of tasks and questionnaires (see Benedek, Könen, & Neubauer, 2012b) the present analyses focus on a detailed time-dependent analysis of free word association behavior at the level of single responses, which has not been previously analyzed or reported.

Word association behavior was assessed by means of a continuous free word association task which required participants to write down all associations for a given stimulus word that come to their mind within a time period of 60 s. Six stimulus words (“Straße” [street], “rot” [red], “König” [king], “Licht” [light], “Berg” [mountain], “Löwe” [lion]) were selected from the German translation of the Kent-Rosanoff word association test (Kent & Rosanoff, 1910; Russell, 1970). Items were chosen to reflect a set of diverse common concepts which are not prone to elicit dominant primary associations according to association norms. The participants were instructed as follows: “Association task: You are now presented six words one after the other. For each word, you have 60 s to write down all things that come to your mind. Your responses should always be single words but not phrases or sentences. Try to find as many different associations as possible.” They were then presented a brief example (“summer”: “beach, sea, warm, swim, sandals, ozone, winter,…”). Finally, they were told that after half-time of each item (i.e., after 30 s) they will be asked to underline the most recent response. This mark was used to obtain the number of responses within the first and second half of the task.

Creativity was assessed with respect to divergent thinking ability, self-reported ideational behavior, and self-reported creativity. Divergent thinking (DT) was assessed by means of two items of the alternate uses task (wine bottle, and compact disk). The task duration was 2 min for each item. The originality of all responses was scored by four independent experienced raters on a 4-point Likert-scale ranging from 0 (“not original”) to 3 (“highly original”; ICC  =; .88 and.86 for item 1 and item 2, respectively). According to common procedures (e.g., Torrance, 1974) the originality ratings of all ideas within a task were added up to compute a total score of divergent thinking. This score thus reflects both, the number of ideas generated by participants (i.e., ideational fluency) and the rated originality of those ideas. Self-reported creative ideational behavior was assessed by means of a German translation of the *Runco Ideational Behavior Scale* (RIBS; Runco, Plucker, & Lim, 2001) employing the 17 first-factor items (e.g., “I have many wild ideas.”). Self-reported creativity (C-SR) was assessed by asking the participants to rate their own general creativity (Kaufman & Baer, 2004) on a 6-point Likert-scale ranging from 1 (“not creative”) to 6 (“highly creative”). To obtain a broad and robust criterion representing different aspects of the multifaceted construct of creativity, the divergent thinking score, self-reported ideational behavior, and self-reported creativity were z-standardized and averaged resulting in a composite measure of creativity (Martindale & Dailey, 1996; Vartanian, Martindale, & Kwiatowski, 2007). The feasibility of this procedure is generally supported by significant inter-correlations of all three measures (*r*[DT, RIBS]  =; .31, p < .001; *r*[DT, C-SR]  =; .17, p < .05; *r*[RIBS, C-SR]  =; .61, p < .001). The composite score showed moderate internal consistency (Cronbach’s α  =; .62).

Finally, groups of low and high creativity (LC vs. HC) were created by selecting the lower and upper 33%; (n  =; 50, each) of the creativity total score distribution. The two groups differed significantly with respect to all three single creativity measures including DT (LC: *M*  =; 2.99, *SD*  =; 1.07 vs. HC: *M*  =; 5.35, *SD*  =; 1.61; *t*[85.10]  =; 8.64, p < .001, *d*  =; 1.44), RIBS (LC: *M*  =; 62.24, *SD*  =; 9.00 vs. HC: *M*  =; 83.38, *SD*  =; 7.38; *t*[98]  =; 12.85, p < .001, *d*  =; 1.82), and C-SR (LC: *M*  =; 3.43, *SD*  =; 0.84 vs. HC: *M*  =; 5.00, *SD*  =; 0.61; *t*[87.14]  =; 10.64, p < .001, *d*  =; 1.69), and, of course, in the total creativity score (LC: *M*  =; −0.86, *SD*  =; 0.46 vs. HC: *M* =;0.79, *SD*  =; 0.31; *t*[85.46]  =; 20.90, p < .01, *d*  =; 2.17), but not with respect to mean age (LC: *M*  =; 21.94, *SD* =;2.34 vs. HC: *M*  =; 22.58, *SD*  =; 3.28; *t*[88.73]  =; 1.11, *ns*), nor sex ratio (LC: 38%; female participants vs. HC: 46%; female participants; χ^2^[1]  =; 0.66, *ns*).

### Analysis Methods

Mednick (1962) conceived the notion of an association hierarchy as the set of most common associative responses to a given semantic concept that can be ranked by their associative response strength. When assuming that creative people show flatter associative hierarchies, this implies that people generally show a similar ranking of associations, but that the gradient of this hierarchy may still differ between groups (see example in Figure1). The *associative (response) strength* of an association response can be estimated by its relative response frequency in free association tasks (Jenkins & Russell, 1952; Nelson et al., 2000). To map the gradient of associative hierarchies for high and low creative people, we, therefore, first identified the general hierarchy (i.e., ranking) of the most common responses in the total sample, and then estimated the associative strengths of these responses for each group separately.

All written responses in the continuous free association task (*n*  =; 13,094 responses) were transcribed to digital media for further computer-based processing. Matlab scripts were used to create a pool of responses for each item and to compute the frequency of occurrence for each discrete response. The associative strength of a response then was defined as the relative response frequency of this response in the continuous association task. For example, if a stimulus word evoked a specific association response in 65%; of participants, this response was defined to have an associative strength of.65. The *uncommonness* of an association was defined as 1 minus its relative response frequency (i.e., associative strength). A frequent response hence has high associative strength and reflects low uncommonness, whereas a rare response has low association strength and reflects high uncommonness.

Associative hierarchies were estimated for the 10 most common associations of the total sample. The analysis was restricted to 10 responses as for this number each response showed an associative strength of.15 or larger; for responses with lower associative strength the estimates might lack reliability as only very few people give these responses (less than 15%;). For example, for the item “Straße” [street], the top-10 responses were “Auto” [car] (72.7%;), “Ampel” [traffic light] (67.3%;), “Asphalt” [asphalt] (46.7%;), “Fußgänger” [pedestrian] (44.0%;), “Zebrastreifen” [cross-walk] (42.7%;), “Unfall” [accident] (32.0%;), “Verkehr” [traffic] (32.0%;), “Autobahn” [high-way] (27.3%;), “Fahrrad” [bicycle] (24.0%;), “Gehsteig” [sidewalk] (20.7%;). Averaging association strengths of the top-10 responses over items then results in an estimate of the average associative hierarchy. As illustrated by this example, association strength measures are usually defined at group level. To be able to compare associative hierarchies statistically between groups, association strengths of the top-10 responses of each item were re-computed within creativity groups in a slightly different way. For each item and every top-10 response, an individual was assigned the score 1 if he had generated this response (e.g., “car” to the item “street”), or 0 otherwise. Averaging these 6 times 10 scores over all six items results in individual estimates of the probabilities to produce the top-10 responses to different concepts. Averaging again over group members precisely returns the average association strengths of the 10 most common association responses (i.e., associative hierarchy) for each group. This procedure hence provides individual scores which can be used for a statistical comparison of association strength between groups. For further tests of Mednick’s hypotheses, the time course of associative fluency and uncommonness was analyzed. At this, the uncommonness of responses was also examined at the level of single consecutive responses.

## Results

### Associative Hierarchies

**Figure 2:**
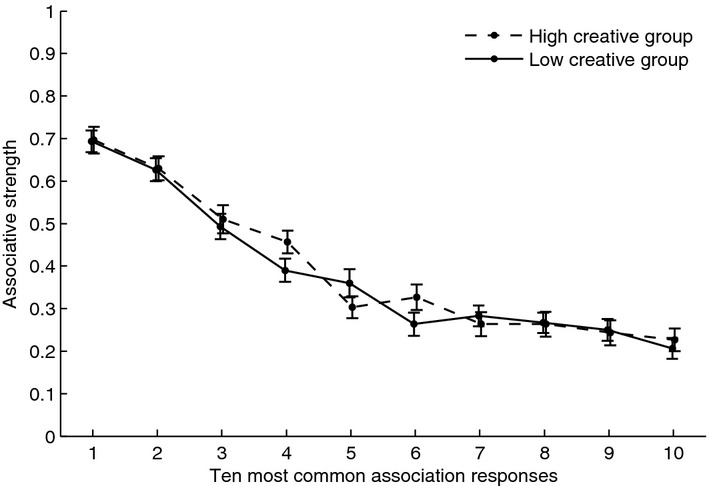
Associative hierarchies of high and low creative people. The association hierarchies reflect the average associative strength [relative response frequency] of the 10 most common association responses to given concepts. Error bars denote 1 standard error of mean.

For a direct test of Mednick’s main assumption, associative hierarchies were compared between groups of low and high creativity. According to this hypothesis, creative people should show lower association strength for highly common responses and higher association strength for less common responses. To test this, the association strength of the 10 most common association responses was analyzed by means of a two-way ANOVA considering the within-subject factor *ranking* (1st to 10th position within a ranking of overall association strength) and the between-subject factor *group* (low vs. high creative). This analysis revealed a highly significant effect of *ranking* (*F*[9,882]  =; 83.33, p < .001, η^2^_partial_  =; .46) indicating that the 10 most common associations differ significantly in their associative strength ranging from 69.5%; down to 21.7%; (see Figure 2). There was, however, neither an interaction (*F*[9,882]  =; 0.98, *ns*) of *ranking* and *group*, as would have been predicted by Mednick’s hypothesis, nor a significant main effect of *group* (*F*[1,98]  =; 0.23, *ns*). As another test, we compared the actual gradients of association hierarchies between high and low creative people. We computed the regression slopes of the individual associative hierarchies and compared the slope parameters between groups. The slopes did not differ between creativity groups (*b*  =; −.052; *t*[98]  =; −0.28, *ns*).

We then examined whether these results are sensitive to the employed criterion for defining creativity groups. To this end, we repeated analyses for groups representing the upper and lower tercile either in the divergent thinking score distribution or in the distribution of the averaged standardized measures of self-reported creativity. The results matched those obtained for the composite creativity criterion: There was no interaction between *ranking* and *group* (*F*(9,882)  =; 0.49, *ns*; *F*(9,882)  =; 1.19, *ns*) and no group difference in slope parameters (*b*  =; −.053, *t*[98]  =; −1.00, ns; *b*  =; −.052, *t*[98]  =; 0.54, *ns*) for groups high and low in divergent thinking or self-reported creativity, respectively. High and low creative people thus were not found to differ in their associative hierarchies reflecting the association strength of the overall 10 most common associations.

### Fluency and Uncommonness of Associations Over Time

**Figure 3:**
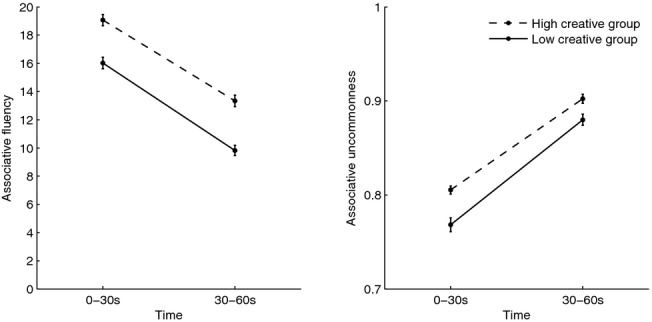
Associative fluency [responses per minute] and uncommonness [1—relative response frequency] in the first (0–30 s) and second (30–60 s) half of the association task compared for groups of low and high creativity. Error bars denote 1 standard error of mean.

To test Mednick’s further hypotheses, the association performance (i.e., associative fluency and associative uncommonness) was analyzed by means of two-way ANOVAs considering the within-subject factor *time* (first vs. second half of task) and the between-subject factor *group* (low vs. high creative). We found that associative fluency generally decreases over time (*F*[1,98]  =; 781.04, p < .001, η^2^_partial_  =; .89), with an average rate of 18 associations per minute (*SEM*  =; 0.29) in the first half of the task to 12 associations per minute (*SEM*  =; 0.27) in the second half of the task (see Figure3). Moreover, high creative people show significantly higher associative fluency (*F*[1,98]  =; 40.73, p < .001, η^2^_partial_  =; .29) generating about 16 associative responses per minute (*SEM*  =; 0.36) as compared to a response rate of 13 (*SEM*  =; 0.36) in the low creative group. There was no interaction of *time* and *group* (*F*[1,98]  =; 1.24, *ns*).

Considering the average uncommonness of associations, we found that uncommonness generally increases over time (*F*[1,98]  =; 455.25, p < .001, η^2^_partial_  =; .82; see Figure 3). Moreover, high creative people generate responses of higher average uncommonness than low creative people (*F*[1,98]  =; 21.15, p < .001, η^2^_partial_  =; .18). There again was no interaction of *time* and *group* (*F*[1,98]  =; 2.30, *ns*). As there had been significant group differences in associative fluency, in a next step, an ANCOVA was computed controlling effects of fluency on associative uncommonness. In this analysis there no longer was a highly significant group effect, but only a tendency toward higher uncommonness of associations in more creative people (*F*[1,97]  =; 3.32, p  =; .07, η^2^_partial_  =; .03). This suggests that the group effect obtained for associative uncommonness may largely be explained by individual differences in associative fluency.

**Figure 4:**
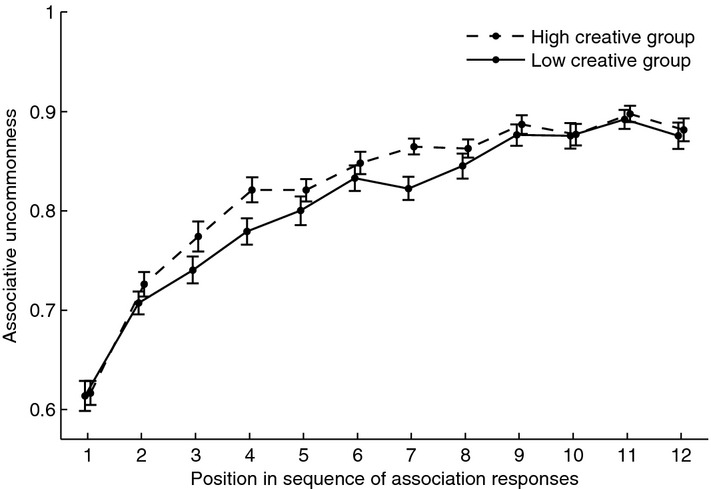
Associative uncommonness [1—relative response frequency] for the first 12 associations within the individual sequence of responses compared for groups of low and high creativity. Error bars denote 1 standard error of mean.

For a more fine-grained analysis of the creativity-uncommonness relationship, we also analyzed the uncommonness of the first 12 associations in the individual sequence of responses. Four individuals from the low creative group had to be excluded from this analysis, because they failed to generate 12 responses in at least one of the association tasks. This analysis again controls for associative fluency by focusing a constant number of responses rather than a constant period of time. An ANOVA was computed considering the within-subject factor *position* (1st to 12th response within the individual sequence of association responses) and the between-subject factor *group* (low vs. high creative). This analysis revealed that uncommonness of associations increases with the sequential position of responses (*F*[9.40,883.79]  =; 98.68, p < .001, η^2^_partial_  =; .51; see Figure4). The primary association thus shows the lowest uncommonness, whereas later associations on average become more and more uncommon (see Figure4). A significant effect of *group* (*F*[1,94]  =; 7.19, p < .01, η^2^_partial_  =; .07) further indicates that the creative group generated more uncommon responses. The interaction of *position* and *group* was nonsignificant (*F*[9.40,883.79]  =; 0.81, *ns*), although the means suggests that this group effect may not be equally valid for all single ordinal positions (see Figure4).

## Discussion

This study aimed at a detailed test of Mednick’s (1962) model on the associative basis of creativity assuming that creative people have flatter associative hierarchies and as a consequence show higher associative fluency (in the long run) and higher uncommonness of associations. We will first discuss the findings related to these three hypotheses separately and then try to draw some general conclusions.

### Creativity and Associative Hierarchies

A direct comparison of the associative hierarchies of high and low creative people did not reveal a significant difference. The association strength of the 10 most common associations was largely identical. This was especially true for the most dominant associations, for which Mednick assumed the largest difference (see Figure1). Moreover, the gradients of association hierarchies did not differ between creativity groups. Maybe, creative people differ only in the association strength of more uncommon responses and differences are more subtle than suggested by the original model. However, highly uncommon or unique responses, per definition, have a very low frequency of occurrence, which impedes a robust assessment and comparison of their (very low) association strengths.

This finding suggests that creative people do not differ in the general organization of their associative memory from less creative people. When asked for an association to “street”, high and low creative people will respond “car” with an equally high probability. The association strength between concepts may be conceived to reflect the contiguity of these concepts in everyday experiences. Association strengths can, for example, be adequately predicted by the relative frequency of co-occurrence of these concepts in written text (Wettler et al., 2005). More and less creative people who share a common environment and who make many common experiences hence establish common associative hierarchies. In contrast, a deviant associative hierarchy might only result from a sustained exposition to an uncommon environment or maybe from cognitive deficits which prevent the learning of common contiguities. Consequently, the present findings could be limited to creativity defined as everyday creativity or little-c creativity, but it remains unclear whether they can be generalized to creative people characterized by highly eccentric lifestyles or by psychopathological traits.

### Creativity and Associative Fluency

Concerning associative fluency, high creative people showed higher associative fluency than low creative people and did so consistently over time. A positive relation of creativity and associative fluency is in line with Mednick’s general hypothesis as well as with previous findings (Levin, 1978; Mednick et al., 1964; Piers & Kirchner, 1971). Originally, Mednick had assumed that creative people would show higher associative fluency only in the long run, when low creative people already have produced dominant associations and have difficulties in retrieving more uncommon associations due to their low association strength. However, Mednick et al. (1964) already observed that creative people show higher associative fluency consistently over time. This finding stood in contrast with the assumption of creativity-related differences in associative hierarchies, but it is consistent with the present finding of no creativity-related differences in associative hierarchies.

High response fluency can be conceived as a major characteristic of creativity. Creativity has even been conceived as a disinhibition syndrome (Eysenck, 1995; Martindale, 1999). Response fluency is reflected in the measure of ideational fluency, which is one of the most commonly used indicators of creative potential (Runco, 2010). Associative fluency was found to be highly correlated with ideational fluency, and also with other indicators of creativity (Benedek et al., 2012b). Moreover, response fluency may not merely be seen as an indicator of disinhibition, but it was also related to adaptive executive processes (Benedek, Bergner, Könen, Fink, & Neubauer, 2011; Benedek, Franz, Heene, & Neubauer, 2012a; Gilhooly, Fioratou, Anthony, & Wynn, 2007; Nusbaum & Silvia, 2011). Cognitive control, referring to the ability to inhibit predominant and irrelevant responses, was found to be positively correlated with ideational fluency (Benedek et al., 2012a). Effective cognitive control may likewise facilitate association fluency (Gilhooly et al., 2007). The effective suppression of salient but irrelevant response alternatives (e.g., common and previous responses) is necessary to avoid perseveration and facilitates the fluent retrieval of new associative responses. In contrast, low cognitive control might cause difficulties to loosen from initial dominant responses, which could eventually decrease response fluency.

### Creativity and Associative Uncommonness

Mednick also assumed that flat associative hierarchies should allow creative people to have more uncommon associations. First of all, we found that uncommonness of association increases over time and over responses. This reflects the well-known finding that initial responses usually are highly common; common responses, however, are soon depleted, so that later responses have to refer to an increasingly extended conceptual range and thus become more and more uncommon (Beaty & Silvia, 2012; Olczak & Kaplan, 1969; Piers & Kirchner, 1971). Considering a constant period of time, high creative people showed a much higher average uncommonness of associations than low creative people. After controlling for individual differences in associative fluency (either statistically using fluency as covariate, or by focusing on a constant number of responses) this group effect, however, nearly disappeared. This suggests that individual differences in uncommonness are largely due to differences in fluency. Because uncommonness increases over responses, those people who are more fluent in retrieving associations will sooner get to retrieve more uncommon associations. This finding hence illustrates an interesting way of how response quantity may affect response quality.

Despite the fact that association fluency may be the main driving force underlying associative uncommonness, there was evidence that even after controlling for fluency high creative people still show somewhat higher associative uncommonness. This may not be true in the very beginning of continuous association (when all people respond with highly common ideas), and maybe not in a late period of the task (when all responses become highly unique), but rather in between. One explanation for this effect may be the observation that high creative people tend to show a higher chaining of associations (Levin, 1978; Benedek et al., 2012b). Focusing on previous responses rather than the actual stimulus word may cause more uncommon associations. The tendency of associative chaining could again be viewed as an effect of higher associative fluency. For those who generate associations at a high rate, preceding responses are highly salient and thus could affect later associations to a larger extent, whereas those who generate at a lower rate may spend more time processing the stimulus concept and thus will rather stick to it.

Considering the above-mentioned issues, the inconsistencies of previous findings might in part be due to specific methods of investigation. Presuming that there are no creativity-related differences in associative hierarchies but rather differences in executive abilities controlling access to semantic memory, one would expect different results depending on the employed method: When investigated for single free or common associations, creative people are expected to show equally common associations; however, when explicitly asking for uncommon responses (e.g., Merten & Fischer, 1999) or when employing a continuous association task without controlling for differences in response fluency, creative people are expected to show higher uncommonness of associations.

### Limitations of the Study

As a potential limitation of this study, the pool of associative responses has not passed a rigorous procedure of lemmatization. Different responses coming from the same word stem hence were considered separately. Lemmatization is a complex linguistic procedure, which might have resulted in an even more precise assessment of association strength measures. Moreover, it should be noted that the computation of association measures was based on the entire set of responses in the continuous association task, and thus it was not restricted to primary associations as it is the case in studies using single association tasks. Considering first associations can not only yield reliable results but also underestimate the set of relevant associations (Nelson et al., 2000). Our association measures hence may differ somewhat from a scoring focusing on primary responses, but by exploiting all available data it may also result in more dependable scores. It should also be mentioned that the results focus on common associations, for which it is feasible to obtain estimates of associative response strength by means of response frequency analysis. High creative people might still show higher association strength at more remote or even unique associations. However, since unique associations are only given by single individuals, it is problematic to estimate association strengths for these responses and compare them between groups of high and low creative people. Maybe different methods (e.g., studies assessing priming effects or speed of processing) could be used to assess association strengths at individual level to further examine this specific issue. Finally, the findings may be restricted to creativity in terms of everyday creativity. It would be interesting to examine whether they generalize to creativity conceptualized by outstanding creative achievements.

## Conclusions

This study revealed clear support for Mednick’s hypotheses that creative people show higher fluency and uncommonness of associations, but not for the underlying model assuming differences in associative hierarchies to be responsible for these differences in association behavior. The findings suggest that high and low creative people show the same general organization of associative memory (i.e., associative hierarchies), and essentially follow the same order of associative recall starting with highly common responses and proceeding with increasingly uncommon associations. As the central difference, more creative people are much faster in creating associative responses and consequently get to retrieve more uncommon associations earlier. It hence could be said that creative people follow a common path to uncommon thought—but they do so at a much higher speed.

High ideational fluency may be conceived as the result of high cognitive control and effective executive functions (Benedek et al., 2012a; Gilhooly et al., 2007). With regard to the debate on the connection of creativity and mental illness (e.g., Fink, Slamar-Halbedl, Unterrainer, & Weiss, 2012; Silvia & Kaufman, 2010), one might see a relation between the higher uncommonness of associative responses of creative people in this study, and the higher associative uncommonness found for people with psychopathological traits (cf., Merten & Fischer, 1999; Rominger et al., 2011). We believe, however, that the cognitive basis underlying the result of associative uncommonness is a different one. Creative people, in contrast to people with mental disorders, may accomplish uncommonness of thought by means of highly functional and adaptive rather than deviant cognitive processes. In other words, creative people are able to produce uncommon thoughts, while psychotic people cannot do otherwise than having uncommon thoughts.

Of course, creativity can hardly be explained by higher associative fluency and uncommonness alone. Mednick (1962) already accounted for the fact that the generation of a creative idea may not only rely on a faster access to associative elements but that it also requires the ability to adequately select and combine these elements to a meaningful solution. A recent analysis of different types of association abilities found that creative people show a higher ability of deliberate dissociation as well as of associative combination (Benedek et al., 2012b). As further relevant factors, creativity was found to be related to a faster assessment and higher evaluation of concept relatedness (Rossmann & Fink, 2010; Vartanian, Martindale, & Matthews, 2009), which again may be helpful to find links between unrelated concepts. Taken together, these associative abilities may fruitfully act together in the generation of creative ideas. Creativity hence may not necessarily imply a special organization of associative memory, but it may rather rely on advanced executive abilities allowing for a highly effective access and processing of memory content.
